# Lessons from history: morbidity of cold injury in the Royal Marines during the Falklands Conflict of 1982

**DOI:** 10.1186/2046-7648-2-23

**Published:** 2013-08-08

**Authors:** Francis St Clair Golden, Thomas James Roose Francis, Deborah Gallimore, Roger Pethybridge

**Affiliations:** 1 Extreme Environments Laboratory, Department of Sport and Exercise Science (DSES), University of Portsmouth, Spinnaker Building, Cambridge Road, Portsmouth PO1 2ER, UK; 2 Diving Diseases Research Centre, Plymouth, UK; 3 Institute of Naval Medicine, Gosport Hants PO12 2DL, UK

**Keywords:** Non-freezing cold injury, Cold sensitivity, Frostbite, Trench foot, Non-battle-related injury

## Abstract

**Background:**

Environmental conditions in the Falklands Conflict of 1982 favoured the genesis of cold injuries. Immediately, post-war, cold injury morbidity and its contributory factors were assessed, in the personnel of UK 3 Commando Brigade (3 Cdo Bde).

**Methods:**

A questionnaire survey of the 3,006 members of 3 Cdo Bde who landed on the islands was conducted within 6–10 weeks of the end of hostilities. Questions included those relating to features of cold injury, body morphology, age, symptoms experienced, past medical history and other possible contributory causes. Additionally, the unit medical team conducted a cursory examination. Data were sent to the Royal Navy Institute of Naval Medicine (INM), where the degree of likely cold injury was broadly classified (‘asymptomatic’ ‘mild’, ‘moderate’ or ‘severe’). A sample (total 109) was then selected at random from each category and subsequently examined and tested at the INM (nerve conduction, photoplethysmography and thermography testing). Forty-seven non-cold exposed sailors acted as a control group. These contemporaneous records have now been identified and interrogated.

**Results:**

Some 2,354 (78%) completed questionnaires were returned, revealing that 1,505 (64%) had experienced symptoms of non-freezing cold injury. The morbidity in the infantry units was significantly greater than that in the support troops (1,051 (76%) vs 454 (46%), *p* < 0.05). No evidence was found to support an influence of a number of factors, commonly believed to have an aetiological role in the production of cold injury. Whilst there was no significant relationship between past history and cold injury morbidity in the brigade as a whole, or within the infantry units alone, an association *was* identified in the collective infantry units (73%) and the support/headquarter units (59%) (*p* < 0.05).

In comparison with uninjured sailors who acted as controls (*n* = 47), nerve conduction was impaired in 35% of those screened some months after returning to the UK, while the photoplethysmography and thermographic responses to a cold sensitivity test showed that most (including those classed by questionnaire as asymptomatic) had residual ‘cold sensitivity’.

**Conclusions:**

Although the passage of time has made retrospective interrogation of historical documents hard, the available data do appear to offer valuable historical and clinical insights. Cold injury affected the majority of those fighting in the cold temperate climate of the Falklands. The overwhelming environmental conditions meant that, for most, a past history of cold injury did not appear to represent a risk factor for subsequent injury, as is the case for less severe conditions. Importantly, even asymptomatic individuals when tested often showed physiological evidence of cold injury—perhaps predisposing them to subsequent elevation in risk.

## Background

### The Falklands campaign

The unexpected invasion of the Falkland Islands by Argentine forces on 2 April 1982 took both British politicians and the UK Armed Forces by surprise. Military advisers were well aware that if the recapture of the islands was to be achieved, speedy action was required before the southern hemisphere winter became established; the logistic trail of 8,000 miles of ocean to traverse created additional difficulties. After a week of frenetic planning activity, the task force sailed for the South Atlantic on 9 April 1982. Given the expected autumnal cold and wet weather conditions in the Falkland Islands and previous experience of warfare in such conditions [1-4], the possibility of significant morbidity from cold injury was a major operational consideration.

Following some preceding air and maritime actions, a successful, amphibious landing of the Royal Marine Commandos, together with representatives of commando-trained army units and the Parachute Regiment, was achieved on 21 May 1982 (for a detailed account of the land campaign, see [5,6]). The nature of the terrain necessitated a ‘wet landing’ for most, in which the lower portion of the body was, in general, immersed from the outset. In the initial 3 days following the landing, when the troops were required to secure the area surrounding the landing zone before moving inland, they were subjected to repeated daily air attacks that necessitated taking refuge, usually in half-flooded slit trenches in the boggy ground. Accordingly, from the outset, they were soaked through with few facilities to dry out until the conflict ended 25 days later on 14 June 1982. During the subsequent land battle, the terrain over which the troops were required to operate varied between marshy bogs, ground covered with sizeable grassy tufts, or steeply undulating rock-strewn ground, to barren mountain slopes [5,6]. Climatic conditions ashore (from contemporary local naval records) throughout the conflict were generally cold and wet (rain, sleet and snow), with a mean ambient temperature around 0.5°C (range +4°C to −3°C), with a minimum of −12°C on the mountains [5] and wind speeds of up to 20.6 m.s^−1^ (74 kph).

### Cold injuries

Cold injuries (CI) can be classified as being either of the freezing (FCI) or non-freezing (NFCI) variety. FCI (synonym *frostbite*) results when intra- and extracellular fluid crystallises at a temperature of −0.55°C and below. NFCI (synonym *trench foot*[2], or, in maritime survivors, *immersion foot*[7,8]) may occur following prolonged exposure to tissue temperatures above freezing and up to about 20°C [9-11]. Such tissue temperatures may be achieved in dry sub-zero environments and in above-freezing conditions (especially when wetness causes cooling by evaporation and can reduce efficacy of insulation by clothing).

NFCI pathogenesis appears to relate to cold-induced peripheral vasoconstriction, which reduces convective heat delivery (by blood) to the region. NFCI risk and severity are directly related to the actual tissue temperature attained and the duration of exposure [11-13]. Risk also arises if core body temperature falls (e.g. where personal insulation is inadequate) and with other factors that might reduce peripheral blood flow. These include advancing age; body habitus; smoking; poor fitness, nutrition or hydration; and acute anxiety. A past history of cold sensitivity or CI may also represent a risk factor for subsequent NFCI.

Clinical presentation is characterised by a history of initial peripheral pain followed by numbness and paraesthesia following exposure. The cooled skin (toes/feet and/or fingers/hands) becomes pale and very cold to touch, with delayed nail bed capillary filling. Peripheral pulses are difficult to palpate. After a relative short period of exposure, particularly in very cold conditions, numbness of the digits, or feet/hands, soon becomes evident and is frequently associated with deep pain and impaired functionality. In some people, tissue swelling may occur after a few hours but more usually after a period of re-warming and the return of a degree of tissue reperfusion (see [10-12] for review of pathogenesis).

Usually, the persistent symptoms of peripheral neuropathy (paraesthesia, numbness and pain) trigger the individual to seek medical advice. Following initial re-warming in less severe exposures, there is frequently an interval of several symptom-free days before the sudden onset of (often nocturnal) peripheral pain, followed by symptoms related to peripheral neuropathy (FStCG clinical experience).

Following less severe exposures, the neurological symptoms generally wane within 8–12 weeks, provided further cold exposure is avoided. Should re-exposure occur before the tissues have fully recovered, the injury will be aggravated and prolonged. Persistent residual signs include pain, hyperhidrosis and some local trophic changes, such as hair loss and nail distortion. In more severe cases [14-16], there is usually a clear demarcation line corresponding with the top of the boot (if it is the foot which is affected), above which the skin may be dusky mottled blue in colour with reduced sensation. Below the demarcation line, the skin is cold, dusky and anaesthetic and may be gangrenous. Peripheral pulses are difficult to palpate, and there can be a total regional loss of sensation, reduced proprioception and even motor paralysis [15]. Subsequently, there is usually a partial return of sensation and motor power, but some muscle weakness and proprioceptive changes may persist. These may manifest as alterations to gait even in the more moderate cases. In very severe cases, trophic changes to the skin and superficial tissues will be present from the outset of treatment that may result in gangrene, necessitating amputation [7,12,15]. Re-warming is associated with marked hyperaemia accompanied by pulsating pain and may require short-term analgesic therapy.

#### Cold hypersensitivity

The great majority of NFCI-injured sufferers experience a persistent cold hypersensitivity [10-12,17], characterised by more intense vasoconstriction on exposure to cold and by slow re-warming and believed to be due to subclinical damage to the peripheral neurovascular tissues. Cold hypersensitivity is also frequently encountered in people with a history of cold exposures but without necessarily having had an overt cold injury. In all cases, re-exposure to cold, before such damage has repaired, may result in a more intense and longer duration of tissue cooling and thus increase both the susceptibility to cold injury and the degree of injury sustained.

#### Historical relevance

Whilst present in many civilian populations during peacetime (e.g. earthquake victims, elderly in poorly heated houses, the homeless sleeping rough and those working or playing in cold environments), the historical incidence of cold injuries can almost reach epidemic levels during wartime (Table 1).

**Table 1:** Reported cold injuries in a variety of conflicts throughout history

Circa 400 BC	Armenia (Xenophon)	‘Cold’ cause of approximately 6,000 (60%) casualties
218 BC	Hannibal crossing the Alps	19,000 (50%) survived from 38,000
1719	Swedish/Norwegian	3,700 Swedish dead from a force of 5,000; 600 permanently crippled from frostbite
1778	American War of Independence	Up to 10% of casualties in some battles
1812	Napoleonic/Russian campaign	100,000 KIA; 200,000 DNBI (majority from cold injury and hypothermia); 12,000 men from the 12th Division all perished except for 350
1854–1856	Crimean War	2,000 cold injured out of 50,000
1861–1865	American Civil War	15,000 cold injury casualties
1870–1871	Franco/Prussian	1,450 CI
1899–1902	Boer War	‘Many with cold injuries’
1904–1905	Russo/Japanese	‘Staggering numbers’
1912	Balkans	‘Many cold casualties’
1914–1918	World War I	British 115,361; French 79,000; Italians 38,000; Germans (number unknown) but had special hospitals dedicated to treating cold injuries (distinction between freezing and non-freezing injury, ‘Trench Foot’, was first made)
1939–1945	World War II	Western Europe: British 500; Americans 91,000
		Italian campaign, winter 1943–1944: British 102 cold injury casualties (ratio 1:45);
		Americans 4,560 (ratio 1:4)
		At sea, ‘Immersion Foot’ was first described
		Russian Front: Germans massive casualties (special cold injury hospitals)
		Attu (Aleutians): US Marines 1,200 in a 15-day period of conflict with a ratio of 1:1 with battle casualties

This is based loosely on [9,14]. The majority of the CI casualties shown probably relate to the more dramatic looking ischaemic extremities, which were blackened and blistered, of FCI. The more innocuous-looking acute NFCI would not have been recognised as such until after re-warming, when pain and swelling resulted in immobility. Thus, the historical incidence of NFCI up to World War I is likely to err significantly on the conservative side.

This paper examines the morbidity of, and possible contributory factors to, NFCI incurred by personnel of the Royal Marine (RM) 3 Commando Brigade (3 Cdo Bde) during the Falklands campaign. The criteria used as the diagnostic marker for NFCI were the presence of symptoms of swelling, peripheral paraesthesia, pain, neuropathy or trophic changes for a period of at least several days. As these signs and symptoms are the preliminary changes seen in the formation of NFCI, it was assumed that, for the purposes of this study, they could be regarded as a marker of NFCI. The paper has been composed from contemporaneous records made in the immediate aftermath of the conflict. Although the passage of time has allowed the work to be published (it was declassified in 2002, and permission to publish was grated), time has also made analysis difficult; we hope the information presented contains valuable historical and clinical insights, especially as those that ignore history (clinical or chronological) are often condemned to re-live it (*after* George Santayana).

## Case presentation

### Methods

Following ethical approval from the MoD Personnel Research Ethics Committee and within 6–10 weeks of their return, a four-page questionnaire, in two sections, was distributed to all 3,006 RM members of 3 Cdo Bde who had landed on the Falkland Islands. The first section related to personal details: onset and duration of symptoms, body insulation used (clothing, footwear, etc.), age, body morphology, smoking habit, nutrition, hydration and past history of CI (defined as having previously experienced symptoms of numbness, pain, swelling, peripheral paraesthesia, neuropathy or trophic changes following a cold exposure). The unit medical personnel completed the second section following a cursory physical examination. A rapid response was encouraged so that the forms could be completed before any symptoms, signs or recollections waned.

Completed questionnaires were returned to the Royal Navy Institute of Naval Medicine (INM) where the data were coded, statistically analysed (ANOVA, Sheffe method of multiple comparisons and Pearson PM correlation; GenStat, VSN International, Hemel Hempstead, UK), and based on reported symptoms, the respondents were placed in one of four broad NFCI categories (‘asymptomatic’, ‘mild’, ‘moderate’ and ‘severe’, based on those devised by Ungley et al. [15]). Those with a past history of symptoms of CI were noted to see if this factor was associated with the incidence of recurrence during the campaign.

A sample (*n* = 109) was selected at random from each category and subsequently tested at the INM using standard clinical lower limb nerve conduction, infrared (IR) photoplethysmography of thumb and great-toe pads and infrared thermography of the feet following warm and cold stimulation. The individuals were also interviewed to establish details of their personal exposure and subsequent symptoms of NFCI. Forty-seven non-cold-exposed sailors of a similar age range but with no previous history of NFCI symptoms acted as the control group and were similarly tested. After the initial sample of 109 was tested, many other marines and paratroopers were seen to determine whether they were fit to undertake winter training in 1983.

**Figure 1:**
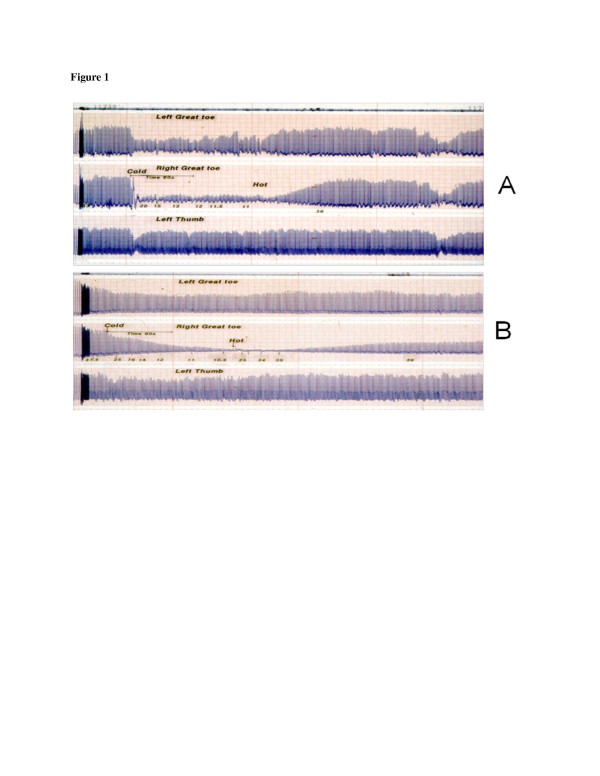
**The photoplethysmograpic blood flow traces of two participants.** An uninjured participant **(A)** and a cold-sensitive Royal Marine **(B)** who still had residual signs of NFCI at the time of testing.

IR photoplethysmographic (Vasculab PPG-13, Medasonics, CA, USA) testing was undertaken with the participants wearing normal underclothing beneath a tracksuit with feet and hands exposed. They were rested in a semi-horizontal seated position in an ambient air temperature maintained in a thermally controlled chamber at 30°C (relative humidity 45%). The IR sensors were attached to the surface of both great-toe pads and one thumb, using clear double-sided adhesive tape. After a 30-min rest, when the pulsatile record was stable, cold water (2°C) from a thermally controlled tank was applied via a standard domestic shower head to the dorsum of one foot for 2 min. This was immediately followed by application of a warm water spray (32°C), continued until such time as the pulse amplitude returned to normal or a 30-min period had elapsed (see Figure 1).

Plantar skin surface temperature was measured using a photographic IR thermal imaging camera (AGA, Lidingo, Sweden). The participants, wearing normal underclothing under a tracksuit, were stabilised at rest in a warm room (30°C) for at least 30 min before they were transferred to a cold environment chamber at an ambient temperature of 10°C. Here they were placed in a pre-warmed sleeping bag (electric torso pad) where they rested horizontally for a minimum of 30 min. During this time, they maintained thermal comfort by self-control of the on/off blanket switch. Following a short period of stabilisation in the thermal comfort zone, both feet were exposed to the ambient cold air—while the rest of the body remained insulated—and an IR thermal image was recorded immediately. After 5 min of cooling in still air, another image was recorded before a forced convective stimulus (electric fan) was applied, to both feet for 5 min. When the fan was switched off, another IR image was taken. Following a further 5 min of continuous exposure of both feet in still cold air, a final image was recorded before replacing the feet in the warm sleeping bag until thermal comfort had been achieved.

### Results

#### Incidence of NFCI

From the total 3,006, for 3 Cdo Bde RM troops deployed on the Falklands, 2,354 (78%) completed questionnaires were returned, of which 2,107 (87%) were received within 3 months of the end of the conflict; the remaining 247 were received over the following three months. The response rate among the RM infantry units (40, 42 and 45 Commando (Cdo)) was 82% (Table 2). An additional 95 responses were received from a number of small separate units who were excluded from the final analysis, as it would have been difficult to make meaningful comparisons with the three major units, *viz*. infantry, Cdo army and RM support unit.

**Table 2:** Questionnaire data showing numbers of questionnaires returned and NFCI morbidity in 3 Commando Brigade personnel

	**RM personnel who landed on Falkland Islands**	**Questionnaires returned (number (%))**	**Presence of symptoms of NFCI (number (%))**
Commando infantry units			
40 Commando	652	398 (61)	314 (79)
42 Commando	494	460 (93)	357 (78)
45 Commando	535	519 (97)	380 (73)
Total	1,681	1,377 (82)	1,051 (76)
Army support units			
29 Cdo Regt RA	396	210 (53)	107(51)
59 Indp Cdo Sqn RE	179	106 (59)	70 (66)
Total	575	316 (55)	177 (56)
RM support units			
CLR	328	328 (100)	87 (27)
Brigade HQ & Sigs Sqn	422	333 (79)	190 (57)
Total	750	661 (88)	277 (42)
Sub-total (all support units)	1325	977 (74)	454 (46)
Grand total	*3,006*	*2,354 (78)*	*1,505 (64)*

Numbers of questionnaires returned were in rounded up percentages. *3 Cdo Bde* 3 Commando Brigade, *29 Cdo Regt RA* Commando Regiment Royal Artillery, *59 Indp Cdo Sqn RE* 59 Independent Commando Squadron Royal Engineers, *CLR* commando logistics regiment, *HQ* &*Sigs Sqn* brigade headquarters and signal squadron.

Of the respondents, 1,505 (64%) appeared to have suffered symptoms of NFCI. Of these, 154 (10%) still had overt residual neurological symptoms 3 months after the conflict ended. The incidence of NFCI in the Cdo infantry units was 1,051 cases (76%), which was significantly greater (*p* < 0.05) than the 177 (56%) cases in the Cdo army support units—consisting of 29 Commando Regiment Royal Artillery (29 Cdo Regt RA) and 59 Independent Commando Squadron Royal Engineers (59 Ind Cdo Sqn RE), and the 277 (42%) of the RM support units—consisting of the Brigade headquarters and signal squadron (HQ & Sigs Sqn) along with the commando logistics regiment (CLR).

In the three Cdo infantry units, 225 men (21%) who reported symptoms of NFCI during the campaign sought medical attention for their symptoms, while only 15 men (8%) did so from the Cdo army units (29 Cdo Regt RA and 59 Ind Cdo Sqn RE) and 17 men (6%) from the RM support units (HQ & Sigs Sqn and CLR). The most commonly reported acute symptoms were persistent numbness (79%), tingling (64%), throbbing (50%), pain (48%), pins and needles (41%) and swelling (28%). Nearly all suffered from blistered feet.

#### Contributory causes

Other than the marked severity of the cold environmental conditions, from the available information, none of the usual contributory causes of cold injury generally referred to in the literature (e.g. personal morphology, smoking habits, nutrition, hydration, insulation including clothing, footwear) appeared strongly related to NFCI risk. Although, from the questionnaire data, there was no significant relationship between past history and cold injury morbidity in the brigade (Bde) as a whole or within the infantry units alone, this association *was* identified in the collective infantry units (73%) and the support/headquarter (HQ) units (59%) (*p* < 0.05).

There was no apparent difference in the incidence of NFCI between the 2,016 (86%) personnel with more than 2 years of service and the 338 (14%) with less than 2 years of service. While 80% of the personnel serving in the infantry units had more than 2 years of military service, as a group they tended to be younger than those in the support units. Again, however, no significant correlation between age and the incidence of CI symptoms was found across all units. In the RM support units (HQ & Sigs Sqn and CLR), there was a decreased incidence of NFCI with increasing age, falling significantly (*p* < 0.05) from 71% of the 139 individuals less than 20 years of age to 55% of the 124 over 30 years of age.

#### Interview results

The interviews of the 109 Falkland veterans, who were seen at the INM in the early months following their return to UK, provided dramatic accounts of their experiences of marching inland to engage with the enemy troops. The difficulties encountered incorporated most of the classical features believed to be responsible in the production of NFCI. The following composite account is illustrative of the experiences of most of those interviewed.

If one fell, it was a major effort to get up, which involved the assistance of several comrades who themselves were struggling under their own appalling loads and fatigue. They ‘yomped’ (marched) all the first day and half that night before bedding down in the open for the remainder of the night. Unfortunately it rained heavily before dawn and, without tents, with only ponchos (waterproofed capes) for protection, they were soaked through. The following morning they set out again but this time took only ration packs and weapons in anticipation of enemy contact.

That night they reached their objective, the small settlement at Teal, where they adopted defensive positions around the settlement. There, they were able to relax and bed down for 2–3 days while ‘recce groups’ surveyed the surrounding countryside. But without the benefit of sleeping bags on a bitterly cold night, many, despite their exhaustion, kept walking in a circle to help maintain body heat. The ambient temperature was below 0°C. At this stage, some noticed numbness of their feet with paraesthesia on weight bearing. Most had blisters. Once contact with the enemy was made, the men moved forward again often through driving rain and falling snow. As the enemy positions were predominantly on the upper regions of the mountains, it was necessary for a series of assaults over steep gradients to occur, frequently at night, often in sub-zero temperatures with very strong winds—survival conditions.

After several days of these horrific conditions, with fluctuating temperatures, precipitation and high wind speeds, many lost all sensation in their now white toes, and paraesthesia made it difficult for some to sleep at night. For these, weight bearing first thing in the morning was particularly painful. On occasions, the weather improved, in that it stopped raining, but [it] froze instead. This pattern continued to be repeated in a series of assaults on the other mountains and hills leading to Port Stanley until the conflict ended on 14th June.

The Commanding Officer reported [5]: There were few places where a handful of men could be brought in to dry out, and once the Commando Brigade was established in the mountains overlooking Port Stanley, [there was] none. There were few tents. Once a man was wet, he stayed wet, unless he could dry his clothes in the surprisingly frequent but usually short periods of sunshine. But hanging out clothes to dry is not a recommended practice in forward positions and difficult on the march.

For the majority of the 25 days of the campaign, their feet were wet and cold. Numbness began to develop after about 2–3 days. Later, in addition to the numbness, they experienced pain and a sensation likened to ‘painful electric shocks running up their legs from their toes’, making sleep difficult despite their fatigue. On weight bearing in the morning, the pain was sometimes almost unbearable for the first 5 or 10 min, but with exercise, this would gradually wane, being replaced by numbness. Some—particularly those with very severe nocturnal pain—found their feet had swollen to such a degree in the morning that they had difficulty in putting on their boots, or if it had been necessary for them to sleep with their boots on, they had difficulty in tying the laces. The 70 most severe cases were transferred to the hospital ship *Uganda*[18]. The majority, however, out of a sense of loyalty to their comrades and a desire to be ‘in-at-the-end’, persevered with remarkable fortitude, although most were hobbling by that time.

Despite the pain, numbness and paraesthesia were gradually spreading proximally; it was not until 24 June 1982, 10 days following the end of hostilities, when they embarked on the ships to come home that many had the time to appreciate the extent of their problem. The pain and numbness led many to kick the ship’s bulkheads repeatedly in an attempt to bring sensation back to their feet. For the majority, sensation gradually recovered during the voyage home and most had a full return of sensation by the time they docked in the UK, just over 2 weeks later, on 11 July 1982 or by the end of their leave in early September.

#### Insulation

Due to the general scarcity of tents, the forward troops were dependent on their ‘ponchos’ for shelter from the rain, but these were only effective on relatively calm days. Consequently, they encountered difficulties in keeping clothing dry, even when resting. In the prevailing conditions, most of the troops never dried out completely [5,6] from the day of landing until after Port Stanley was captured, when wet clothes could be hung out to dry.

Surprisingly, for a uniformed service, 46 different types of boot were worn. The majority of troops (75%) wore one of two standard issues (General Service (GS) or Cairngorm Mountain Boots). However, there was no significant difference in the incidence of NFCI between any of the boot types worn. Regardless of which boot was being used, between 50% and 70% wore more than one pair of socks at any one time. However, the conditions were such that it was not possible to dry spare pairs of socks adequately between use. Meshed plastic insulating insoles were used by 80%.

#### Nutrition and fluid intake

The daily food ration came from standard British Army ration packs. These were of two types: the most common was the GS basic ration pack, composed largely of tinned foods with an energy content of 16,830 kJ (4,022 kcal)/ 24 h, and the arctic ration pack, composed of dehydrated food and powders for drink mixes, with an energy value of 18,950 kJ (4,529 kcal) [19]. The higher calorific content of the latter was designed to meet the increased energy demands of cross-country skiing and pulk (sledge) hauling, etc. When available, the arctic pack was preferred by the troops to the GS pack (90% *cf*. 64%) as the contents were less bulky, lighter and more nutritious. However, as they required just over a litre of water to reconstitute, they often proved unsuitable.

The lack of adequate, reliable data on the actual intake of food and fluid, daily energy expenditure and consequent energy balance made an analysis of these risk factors for NFCI impossible. It was noteworthy, however, that all the front-line troops constantly complained of feeling hungry, and they frequently consumed enemy rations when caches were discovered in vacated positions. The shortage of transport helicopters coupled with the speed of advance of the troops resulted in difficulties in re-supply. This produced a degree of randomness in the availability of food and fluid. The poor availability of potable water occasionally caused a problem. Although there was a plentiful supply of rain, its collection was almost impossible while on the move or during combat. On those occasions when re-supply was possible, extra ammunition took priority over bottled water. Although ground water was in abundance, purifying it proved problematic, while on occasions, freezing of the filter bags made it impossible without the ability to thaw.

#### Physiological testing

Prior to testing the 109 individuals who had been randomly selected from the four identified sample groups, closer questioning and physical examination revealed that most of those who had classed themselves as asymptomatic, from the questionnaire analysis, had suffered NFCI.

In 35% of the RM personnel tested, there was a range of impaired nerve conduction of the peripheral sensory nerves of the lower limbs. In some, the results suggested damage that was more extensive than the subjective symptoms would suggest. Even in some of those in asymptomatic category, without residual symptoms, were found on clinical examination to have mild peripheral nerve damage. All of those in the control group (*n* = 47) were found to be normal in this respect.

In general, the plethysmographic testing in those injured showed evidence of marked cold sensitivity in most cases, characterised by a delay both in the onset and rate, of vasoconstriction compared to those in normal controls. On re-warming, again, unlike the response of the controls, there was a marked delay in the rate of return of the pulse amplitude to control values; typical examples of these responses are presented in Figure 1.

**Figure 2:**
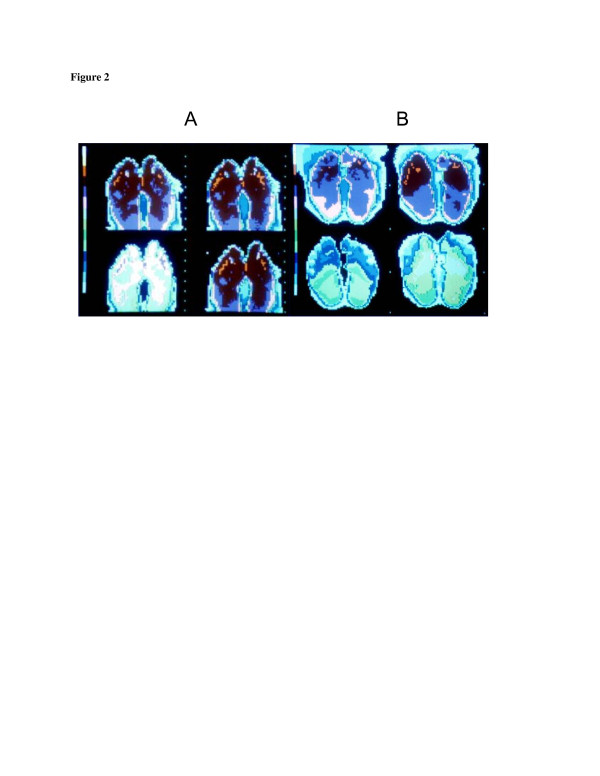
**Thermographic feet images of uninjured participant (A) and NFCI patient (B) to cold air (10°C).** Thermal scale on *left side* of picture: *white*/*pale blue* = coldest and *red* = warmest. *Top right image*: on initial exposure to cold air. *Top left image*: after 5 min of exposure and just prior to forced convective stimulus. *Bottom left image*: at the end of a 5-min forced convective stimulus. *Bottom right image*: at the end of a further 5 min of exposure to still air.

In this figure, trace A is the result from an uninjured individual who demonstrates an immediate vasoconstriction response to the cold stimulus in both toes, with that in the stimulated right foot being more pronounced. There is a corresponding, short-duration reflex constrictor response in the thumb. Following 2 min of cooling, the re-warming stimulus is commenced and the pulse amplitude returns to control levels within approximately 2 min. Trace B is from a cold-injured RM with residual symptoms; the rate of onset of vasoconstriction is slow but intense. Although the reflex constrictor response in the thumb is present, it is much less marked than that seen in uninjured individuals. On re-warming, the amplitude of the signal has not returned to the resting level after half an hour (only the first 4 min are shown in the figure). In the non-cold-stimulated contralateral foot, a cold vasoconstrictor response is present but both delayed in onset and less marked than that in uninjured individuals (Figure 2).

The IR photographic thermography testing revealed a common feature in all of those with histories of severe NFCI: a reduced skin temperature of the plantar surface of the feet and toes just before the cold stimulus commenced, despite the whole body-warming measures that had been taken. The uninjured controls also showed some cooling, which occurred in the time between the feet being removed from the sleeping bag and positioned for the first picture (approximately 1 min). In these controls, the temperatures of the feet never fell to the level reached by those with a history of NFCI.

At the end of the 5-min convective cooling period, when the fan was switched off, all showed varying levels of cooling, with the cold injured much more visible than the controls. After the 5-min recovery period, the controls showed some degree of re-warming, while in all of those with a history of NFCI, there was a marked reduction in re-warming rates, with the majority still not back to the pre-cooling level by the time the test was concluded. However, approximately 15% of the controls also displayed a cold sensitivity response at a level that overlapped the 5–10 percentile of the CI group.

### Discussion

The principal objective of the manuscript is to report the burden of NFCI incurred by 3 Cdo Bde personnel during the Falklands campaign of 1982. A secondary objective was to report possible contributory factors, with the view to learning lessons that could prove to be of value in increasing the understanding of the condition. Such knowledge may also be beneficial to future RM personnel selection, training policies, operational procedures in cold climates and matters relating to the management of injured personnel.

Those who experienced acute symptoms of either numbness, tingling, throbbing, pain, pins and needles, blistering or swelling—which were attributed to peripheral neuropathy and cold-induced vasomotor changes—were classified as having NFCI. However, the ‘blistering’ recorded by most as occurring during the second or third day of the march inland was considered more likely due to abrasion of oedematous skin with water-sodden socks, possibly aided by some postural tissue swelling, rather than cold injury.

From the returned questionnaires from 3 Cdo Bde as a whole, NFCI appeared to have affected 1,505 (64%), with the highest incidence found in the infantry units (76%, 1,051 cases). The morbidity in the support units was 177 cases (56%) in 29 Cdo Regt RA and 59 Ind Cdo Sqn RE, and 277 cases (42%) in the HQ & Sigs Sqn and CLR. Thus, the morbidity in the infantry was greater than in the support units. Previously published UK casualty statistics for the campaign [18], gave the CI morbidity as 70 individuals, i.e. 13.6% of the total number of combat casualties evacuated to the hospital ship Uganda. However, this figure relates only to those who were immobilised and does not reflect the overall numbers with less severe cold injuries reported in this paper, and who ‘soldiered on’, although some did seek medical attention (16% infantry, 5% RM support units and 2% army support units).

The 82% questionnaire response rate from the infantry units (40, 42 and 45 Cdo), who were the fighting teeth of 3 Cdo Bde RM, and thus the most likely to suffer CI, was also commendable. Regrettably, while the response from both 42 and 45 Cdo was excellent (93% and 97%, respectively), that from 40 Cdo was only 61% (Table 2). This discrepancy could lead to an underestimation of the true morbidity in the Cdo infantry units. Given the similarity in environmental conditions for all 3 Cdo infantry units, had the questionnaire response rate of 40 Cdo been similar to those from 42 and 45 Cdo, it is likely that this would have increased the 40 Cdo questionnaire response rate from 398 to 597 and their morbidity from 314 to 464 cases. This increased response would also have raised the overall total NFCI morbidity for the infantry units from 1,051 to 1,201 but, obviously, leave the percentage rate unchanged (76%). The overall morbidly for 3 Cdo Bde, likewise, would also have been only marginally increased by 1% to 65%. Given the conditions, had the response rate of the entire Bde been better, the overall morbidity would also have been higher.

Another potential cause of underestimation of morbidity was the under-reporting of symptoms. Of those who had classed themselves as being asymptomatic in the questionnaire and were subsequently selected in a random sample for examination at the INM, most were found to have been cold injured. Should that rate of under-reporting be common throughout the Bde, again, the overall total morbidity would then have been considerable larger.

Supporting evidence of an expected role by the suspected contributory aetiological factors, excluding past history was unobtainable. Under normal circumstances, the difficulties in collecting high-quality scientific data in the field relating to quantifiable food and fluid intake, precise levels of insulation used, exact timing of events, exercise levels etc. are well understood. In combat conditions, this becomes impossible. In any case, it would appear that throughout the 25-day campaign, the environmental conditions were so severe and had such an effect on peripheral tissue cooling that all possible variables other than past history were overwhelmed. Even then, and somewhat surprisingly, a history of cold injury had less of an influence than might have been expected. While there was no significant relationship between past history and cold injury morbidity within either the Bde as a whole or individual units, this association was identified in the collective infantry units and the support/HQ units.

While, from an epidemiological viewpoint, the high morbidity rate is of interest; perhaps of greater interest should be the reason why 849 (36%) escaped injury in similar environmental circumstance to those injured, in particular, the relatively large number (326 cases, 24%) of infantry personnel who reported as asymptomatic. A possible explanation could be that some, in fear of being medically discharged, intentionally concealed their symptoms. More likely, they misinterpreted the significance of their symptoms, assuming that such sensations, being similar to those they always experienced in the field in cold weather, were the normal default position. Regardless of these suspicions, those asymptomatic cases that were subsequently found to have had NFCI, when examined and tested at the INM, were not added to the questionnaire results shown in Table 2.

The fact that only 10% of those with NFCI stated they still had residual symptoms 3 months after the campaign is of interest. Such a result may at first be reassuring in terms of potential recovery rates and future employability. However, the presence of residual cold sensitivity in the asymptomatic group, who had been physiologically screened at the INM, is less reassuring. This could suggest that there may be many more who were not screened, who considered themselves asymptomatic, but who nevertheless may have longer-term cold sensitivity, thereby increasing their susceptibility to future cold injury. Alternatively, it is possible that the tests used at the INM were oversensitive and as a consequence produced some false positive results. The finding of a cold-sensitive response in approximately 15% of the uninjured control group used in the study could be interpreted as an indication of oversensitivity. If so, the test lacks the necessary specificity for use in isolation as a diagnostic tool [16]. Alternatively, it could mean that in the general UK population, there is a background cohort of cold-sensitised people who have been cold injured as a consequence of participating in some routine activities such as outside winter employment, outdoor social or sporting activities—the very characteristics usually sought in RM pre-joining selection. Regardless, given the importance of establishing whether cold sensitivity is an endemic problem to the RM, and infantry troops in general, it is necessary to establish a scientifically proven methodological technique to test for and quantify the level of cold sensitivity. Under normal research circumstances, the preparation of assessment protocols to carry out such investigations would be developed to vigorous scientific standards well in advance of commencing data gathering. However, the plethysmography and thermography tests, used at the INM, were of necessity devised in haste, specifically to conduct tests on the Falkland veterans as soon as possible after their return to the UK. Work continues to the present time to try and refine these tests [20].

Other studies have identified a background level of cold sensitivity in those regarded as uninjured controls undertaking tests similar to that presented here [16,20]. This raises the questions of whether RM personnel should be routinely screened for cold sensitivity before recruitment, whether cold sensitivity is a common sequela of basic or winter training and what level of cold sensitivity must be reached before it becomes operationally significant. Although these questions could have important implications for the potential exposure limitations of cold-sensitised troops, they are best answered by a longitudinal study employing sensitive and reliable techniques that enable accurate prognostic declarations. From a clinical viewpoint, the descriptions of many of the cold injuries reported, together with the relatively rapid recovery times, would classify them as mild/moderate. However, from a military operational perspective, by the end of the conflict, these injuries had become critical [5].

#### Military lessons

The potential effect of cold injury on the operational efficiency of combat troops fighting in cold wet environments is well understood by military commanders, (e.g. ‘The most serious menace confronting us today is not the German army, which we have practically destroyed, but the weather…may well destroy us through the incidence of Trench Foot’. Lt. Gen. George Patton, 1944). Its frequent recurrence in cold weather conflicts (Table 1) may erroneously be interpreted as a failure of command to plan appropriately, either through ignorance or wilful disregard.

In the severe conditions encountered in the Falklands, the implementation of normal preventative measures was impeded by extraordinary operational requirements that resulted in an incremental daily rise in overt cold injuries. As a consequence, it was not possible to implement many of the lessons learned in this respect from World Wars I and II [3], *viz*. frequent troop rotation, adequate insulation and ready availability of food and warm drinks.

Military planning involves risk assessments of a number of factors that could have consequences to the outcome of a battle or even campaign. In cold environments, the possibility of significant attrition from cold injury is one such factor. However, unlike traumatic ‘battle casualties’, the time of onset to the occurrence of cold injuries often provides some leeway in which to achieve a specific objective before the degree of morbidity reaches a level where the fitness of fighting troops may no longer be adequate to provide an effective fighting force. At the closing stages of the Falklands War, that point was not far off [5].

The personnel of 3 Cdo Bde were elite troops who are experienced in the necessary fieldcraft required to fight and live in cold environments. Therefore, the high NFCI morbidity which occurred should not be regarded as an indication of lack of skill or experience but a reflection of the severity of the prevailing environmental conditions (and lack of water, food and shelter) they had to endure. Not surprisingly, the threat was greater for the men in the forward infantry units (40, 42 and 45 Cdo), who faced continuous exposure to a cocktail of known risk factors, *viz*. extreme weather (wet, windy, intermittent freezing), compromised personal insulation, high energy expenditures with insufficient food intake, long durations of continuous exposure, minimal effective shelter, inadequate effective rest periods (i.e. removal from the adverse climatic conditions for sufficient duration for peripheral tissue temperature re-warming to occur before re-exposure) and the almost unremitting stress of combat. While the level of exposure may have been slightly less for the men in the supporting units, the 49% incidence of NFCI, even in those men, is an indication of the cold stress they also endured. Matters were not helped by the reduced availability of adequate logistic helicopter support in the Falklands, largely as a result of the loss of the logistic support ship *Atlantic Conveyor*.

While winter field training is essential to provide the necessary skills to live and fight in cold conditions, the ever-present associated risk is that this training may result in chronic pathophysiological changes and hypersensitivity to cold which increases susceptibility to future injury. Ideally, peacetime field training exercises are usually of relatively short duration (<5–6 days) with interspersed recovery breaks, particularly if conditions are poor. Accordingly, there is some respite from cold exposure during which the troops can rest, dry out their clothing and enjoy some hot food and drinks. In peacetime exercises, when conditions deteriorate, command measures are taken routinely to reduce exposure levels. Regardless of such safety measures, a small number of Royal Marines periodically experience the early stages of NFCI [21] during winter training. Accordingly, it would not be surprising to find varying levels of cold sensitivity in a cohort of men who, during an extended cold exposure, may succumb to cold injury at different stages. This could explain why, in combat, there is a progressive increase in morbidity levels with time [3]. Ironically, the more realistic peacetime exercises happen when there are greater chances of participants sustaining cold sensitisation and thus increased susceptibility to cold injury.

Cold sensitivity, clearly, is a serious threat to the functionality of fighting troops; therefore, its prevention is worthy of respect in training. This is a difficult decision for commanders who operate a policy of ‘train hard, fight easy’. However, given the known risk of incurring cold sensitisation and NFCI in training, every effort should be taken to avoid its occurrence. It should not be necessary to damage an individual in the process of learning the necessary basic skills of living and fighting in cold environments, especially if at the same time you are compromising future operational capability: cold-sensitised troops, especially in elite units, may be a liability to the success of a mission if tactics have to be altered to cater for cohorts who are already cold sensitised or developing NFCI.

A policy of introducing frequent breaks in training to foster recovery from possible early peripheral neurovascular damage and thus avert the possibility of developing cold sensitivity is recommended. For such a policy to be successful, trainers would require to fully understand the criticality of it. As experience is gained, exposure times could be extended to increase confidence levels and skills. In advanced specialised training, obviously it will be necessary to intensify the duration of exposure.

The nature of the thermal stress confronting infantry and support troops is not simply related to environmental temperature alone; differences in duration of exposures, in addition to other indirect thermoregulatory factors, e.g. ready availability of functional insulation, energy output, work/rest cycles and caloric intake [21,22], can have a very important influence on the outcome. In this respect, infantry can be at a severe disadvantage to support units. The complaint by frontline troops of constantly feeling hungry [6] suggests that their energy input was insufficient to balance their energy output. The subsequent report by the field commanders [5,6] that all of the men lost weight is not surprising. If they had been hypoglycaemic, this would add to the thermoregulatory threat through associated reduction in shivering intensity [22,23].

## Conclusions

NFCI has a long history of producing pathophysiological changes that can have lifelong implications for the health and well-being of the sufferer. In the military context, NFCI can make the difference between operational success and failure. Despite its obvious importance, NFCI remains poorly understood, definitive diagnostic tests await development and important scientific studies await completion. Whilst this situation remains, history has every chance of repeating itself.

## Consent

### Availability of supporting data

Regrettably, the raw data generated during the physiological assessment of RM personnel and controls in 1982 and 1983 were destroyed some time after the surviving authors left the Survival and Thermal Medicine Department of the Institute of Naval Medicine. The data reported in this paper have been compiled from those given in the executive medical report made shortly after the data used were aggregated. Written informed consent was obtained from patients for the publication of their anonymised data, including images.

## Abbreviations

3 Cdo Bde: 3 Commando brigade royal marines; 29 Cdo Regt RA: 29 Commando regiment royal artillery; 59 Ind Cdo Sqn RE: 59 Independent commando squadron royal engineers; Bde: Brigade; CI: Cold injury; Cdo: Commando; CLR: Commando logistics regiment; DNBI: Disease and non-battle-related injury; FCI: Freezing cold injury; GS: General service; HQ: Headquarters; HQ & Sigs Sqn: Headquarters and signal squadron; INM: Institute of naval medicine; IR: Infrared; NFCI: Non-freezing cold injury; RM: Royal marine.

## Competing interests

The authors declare that they have no competing interests.

## Authors’ contributions

FSCG was responsible for the concept and design of the survey of RM personnel after the Falklands Conflict. He undertook the interviewing and medical examination of those who were selected for testing at the INM, in addition to conducting the IR photoplethysmography testing, analysis and production of the report. TJRF conducted the IR plethysmographic testing and analysis as well as co-authoring the report. DG was responsible for the questionnaire data processing and analysis, in addition to drafting the report. RRJP provided advice on the questionnaire design and statistical analysis. FSCG and TJRF were responsible for the preparation of this report. All authors read and approved the final manuscript.

## Authors’ information

FSCG (MB, BCh, Dip AvMed, PhD) is a retired Surgeon Rear Admiral of the Royal Navy and is currently a consultant to the Extreme Environments Laboratory, Department of Sport and Exercise Science (DSES), University of Portsmouth, Spinnaker Building, Cambridge Road. Portsmouth. PO1 2ER UK. TJRF (BSc, MB BS, MSc, PhD, Dip DHM, MFOM, FUHM) Is a retired Surgeon Commander of the Royal Navy, is currently in medicolegal practice in diving medicine and is a faculty member on the Level 2a course in diving medicine for medical practitioners at the Diving Diseases Research Centre, Plymouth, UK.
